# Classifying Tremor Dominant and Postural Instability and Gait Difficulty Subtypes of Parkinson’s Disease from Full-Body Kinematics

**DOI:** 10.3390/s23198330

**Published:** 2023-10-09

**Authors:** N. Jabin Gong, Gari D. Clifford, Christine D. Esper, Stewart A. Factor, J. Lucas McKay, Hyeokhyen Kwon

**Affiliations:** 1 School of Computer Science, College of Computing, Georgia Institute of Technology, Atlanta, GA 30332, USA; ngong6@gatech.edu; 2 Department of Biomedical Informatics, School of Medicine, Emory University, Atlanta, GA 30322, USAlucas@dbmi.emory.edu (J.L.M.); 3 Department of Biomedical Engineering, Emory University and Georgia Institute of Technology, Atlanta, GA 30322, USA; 4 Jean and Paul Amos Parkinson’s Disease and Movement Disorders Program, Department of Neurology, School of Medicine, Emory University, Atlanta, GA 30322, USA; cedoss@emory.edu (C.D.E.); sfactor@emory.edu (S.A.F.)

**Keywords:** machine learning, kinematics, motion capture, Parkinson’s disease, motor subtypes

## Abstract

Characterizing motor subtypes of Parkinson’s disease (PD) is an important aspect of clinical care that is useful for prognosis and medical management. Although all PD cases involve the loss of dopaminergic neurons in the brain, individual cases may present with different combinations of motor signs, which may indicate differences in underlying pathology and potential response to treatment. However, the conventional method for distinguishing PD motor subtypes involves resource-intensive physical examination by a movement disorders specialist. Moreover, the standardized rating scales for PD rely on subjective observation, which requires specialized training and unavoidable inter-rater variability. In this work, we propose a system that uses machine learning models to automatically and objectively identify some PD motor subtypes, specifically Tremor-Dominant (TD) and Postural Instability and Gait Difficulty (PIGD), from 3D kinematic data recorded during walking tasks for patients with PD (MDS-UPDRS-III Score, 34.7 ± 10.5, average disease duration 7.5 ± 4.5 years). This study demonstrates a machine learning model utilizing kinematic data that identifies PD motor subtypes with a 79.6% F1 score (N = 55 patients with parkinsonism). This significantly outperformed a comparison model using classification based on gait features (19.8% F1 score). Variants of our model trained to individual patients achieved a 95.4% F1 score. This analysis revealed that both temporal, spectral, and statistical features from lower body movements are helpful in distinguishing motor subtypes. Automatically assessing PD motor subtypes simply from walking may reduce the time and resources required from specialists, thereby improving patient care for PD treatments. Furthermore, this system can provide objective assessments to track the changes in PD motor subtypes over time to implement and modify appropriate treatment plans for individual patients as needed.

## 1. Introduction

Parkinson’s Disease (PD) is a progressive neurodegenerative disorder caused by degeneration of dopamine-producing neurons in the brain [1]. More than 10 million people are living with PD worldwide, and the incidence of PD is observed to increase with age, where over four percent of people with PD are diagnosed before age 50 [2]. PD prevalence has also been found to be increasing, with the number of people with PD doubling from 1990 to 2015 [3]. PD exhibits motor symptoms including tremor, bradykinesia, rigidity, gait and balance problems, and freezing-of-gait (FOG). Additionally, PD exhibits non-motor symptoms including cognitive impairment, sleep disorders, psychiatric symptoms, and other symptoms that can significantly impact the quality of life for patients [4]. 

PD has a complex disease presentation, and it has been suggested that it is comprised of at least two partially overlapping motor subtypes—tremor dominant (TD) and postural instability/gait difficulty (PIGD) [5]. These two phenotypes are clinically different and, by definition, PD patients with PIGD subtypes exhibit more deficits in balance and gait as compared to the patients with TD subtypes [6]. Proper identification of motor subtypes earlier in the disease course is important as it can help predict progression of the disease and determine appropriate interventions to manage the progression of PD [7]. Compared to TD patients, patients with PIGD are known to exhibit a faster disease progression with greater motor impairment, including a higher risk of falling, worse postural control, and a greater likelihood of experiencing FOG. Treatment for the PIGD subtype is also more challenging with a reduced response to levodopa and surgical intervention with deep brain stimulation therapy [8,9]. Also, motor subtypes can change over the course of PD progression, where a change from TD to PIGD is more likely [10]. Therefore, recognizing and tracking motor subtypes in PD can potentially inform care plans, particularly in low-resource environments where access to movement disorders specialists is limited and as shortfalls of neurologists are projected to worsen [11,12]. 

Conventionally, assessing the motor subtypes of PD is done through expert observation by a movement disorder specialist based on Movement Disorder Society-Unified Parkinson’s Disease Rating Scale Part 3 (MDS-UPDRS-III) measures from standardized movement assessments during movement tasks designed to measure various motor symptoms, including tremor, rigidity, bradykinesia (slowness of movement), and postural instability [5]. Despite a standardized assessment to quantify motor state, the MDS-UPDRS-III may still be somewhat subjective, even when performed by expert clinicians [4,13]. To avoid subjective MDS-UPDRS-III scoring as a gold standard measure of movement, at least two trained movement disorder specialists should examine the patient with inter-rater reliability over 90% [13,14]. When having multiple raters, assessment results can be further improved with techniques like Bayesian aggregation [15]. However, employing multiple raters is very costly with limited resources and accessibility to medical services globally [16,17]. 

For consistent and reliable screening of PD, multiple reports have proposed objectively quantifying various movement features from 3D kinematic data using machine learning or statistical analysis approaches [18,19,20,21]. Amongst them, several works proposed quantifying TD and PIGD subtypes, specifically [6,22,23,24,25]. These analyses include gait patterns from on-body inertial measurement sensors [6,22], frequency component features from standing center of pressure (COP) time-series data [23], demographic and clinical features related to patient falls [24], and even hand-engineered features from resting-state functional magnetic resonance imaging [25]. Although effective for distinguishing PIGD and TD phenotypes, previous studies had two significant limitations. First, they focused on a single aspect of motor phenomena, such as gait, falls, or postural instability, while PD motor symptoms are essentially whole-body phenomena with complex symptoms. Second, most works did not evaluate the bias related to FOG features when quantifying PIGD [6,22,23]. Because patients with PIGD subtypes experience FOG more frequently, it is possible that classifiers designed to identify movement features associated with PIGD were actually biased toward movement features associated with FOG, which is only a part of the PIGD phenotype. Yet, a classifier needs to be unbiased so that it can identify PIGD features for patients not experiencing FOG. Previous studies have explored quantifying movements in FOG in PD using machine learning approaches [26,27,28]. Those works showed that frequency features, such as freeze band, or cycle-to-cycle variation in gait parameters, are effective for quantifying FOG [29,30,31]. It is yet unclear how movement features relating to FOG are associated with distinguishing between TD and PIGD. 

In this work, we propose a system that uses machine learning models to automatically and objectively identify some PD motor subtypes, specifically TD and PIGD, from 3D kinematic data recorded during walking tasks for PD patients recruited from the clinical service of one of the coauthors (MDS-UPDRS-III Score, 34.7 ± 10.5, average disease duration 7.5 ± 4.5 years). We demonstrate that optimally distinguishing between TD and PIGD in PD requires analysis of full-body kinematics, emphasizing the whole-body nature of PD symptoms. Additionally, we investigated the minimal set of instrumentation necessary for the task. Our analysis on a minimal instrumentation set focused on the lower limbs provided reasonable performance, which provided an opportunity to deploy a wearable-based system with a reduced subject burden. 

In our experiment, we analyzed movements during standardized walking and turning tasks for patients including advanced PD experiencing FOG [32] and Hoehn and Yahr (H&Y) Stages of II (N = 43), III (N = 8), and IV (N = 8). In order to approximate the “state-of-the-art”, we compared our model based on temporal-frequency features derived from full-body kinematics to a comparable model based on established gait-based temporal features. We found that when used to classify TD and PIGD, our model significantly outperformed the comparable gait-feature-based model (absolute 22.3% F1 score increase relative to 57.3% F1 score of the baseline) for PD patients with a FOG score of 0, which is item 3.11 from the MDS-UPDRS III, demonstrating that our model is not being biased toward movements relating to FOG. Further, our analysis showed that temporal-frequency features from the lower body movements are particularly useful for distinguishing TD and PIGD. We expect our analysis to objectively quantify TD and PIGD subtypes through novel movement biomarkers to describe the associated movements when patients are walking. Our objectives are twofold: (1) test the ability of different machine learning (ML) models to discriminate TD and PIGD subtypes and (2) test the contribution of particular kinematic marker signals to discrimination performance. 

## 2. Materials and Methods 

### 2.1. Movement Testing 

We collected whole-body 3D kinematics data recorded during comprehensive clinical behavior testing for individuals with PD while in the “OFF” medication state, defined as >12 h after the last intake of all antiparkinsonian medications. 

#### 2.1.1. Study Participants 

This study was registered through clinicaltrials.gov (NCT02387281). The participants were recruited from Emory Movement Disorders Clinic and we were provided with written informed consent according to procedures approved by Emory University IRB. The total number of study participants was N = 56, including individuals diagnosed with PD with and without FOG (N = 51) and individuals with primary progressive freezing of gait (PP-FOG; N = 5), based upon clinical evaluation by a movement disorder specialist. We acknowledge that MDS-UPDRS evaluation from a single movement disorder specialist is a limitation of the study. Despite this, the labels provided were from a movement disorders specialist (SAF) who has used and taught the use of the UPDRS-III and subsequent MDS-UPDRS-III since their initial introduction and who routinely provides similar labels for therapeutic clinical trials. Therefore, we regard these labels as very high quality, notwithstanding that they are from a single rater. 

The inclusion criteria were as follows: Age ≥ 18 years; PD diagnosis according to the United Kingdom Brain Bank criteria [33]; Hoehn and Yahr stage I–IV in the OFF state; willing to participate in every aspect of the study and able to sign a consent document. We referred visual inspections by a movement disorder specialist to confirm participants with FOG [34]. The exclusion criteria were the following: prior treatment with medications that can cause parkinsonism; other neurological or orthopedic disorders interfering with gait; other medical conditions, such as dementia, precluding study completion. Table 1 shows the demographic and clinical characteristics of study participants. PD-FOG represents the patients with PD and FOG, PD-NoFOG represents the patient with PD, but without FOG, and PP-FOG represents patients with other parkinsonism with primary progressive FOG.

#### 2.1.2. Movement Assessment Protocol 

Study participants were assessed with MDS-UPDRS-III motor exam [4] in the “OFF” medication state. Performance was scored in person, and video recordings were completed and used to assess freezing if necessary. Within the MDS-UPDRS-III motor exam, we use three different sessions with three replicates each, including timed-up-and-go tests (TUG) [35], walking straight without turns (Walk-Thru), and turning 360° in place (Turn-360°) for quantifying motor subtype. In our dataset, the average recording durations were 4.75 sec for Walk-Thru sessions, 63.9 sec for Turn-360°sessions, and 86 sec for TUG sessions. Although the testing paradigm was designed to minimize fatigue, we acknowledge the potential for fatigue during testing to have impacted gait impairments in some manner. Based on previous literature [36], we believe it is most likely that the effects of Parkinson’s disease were considerably stronger than the effects of fatigue. For example, gait features in Parkinson’s disease even in the ideal ON medication state are relatively far from comparable measures from healthy controls. 

#### 2.1.3. Motion Capture 

**Figure 1:**
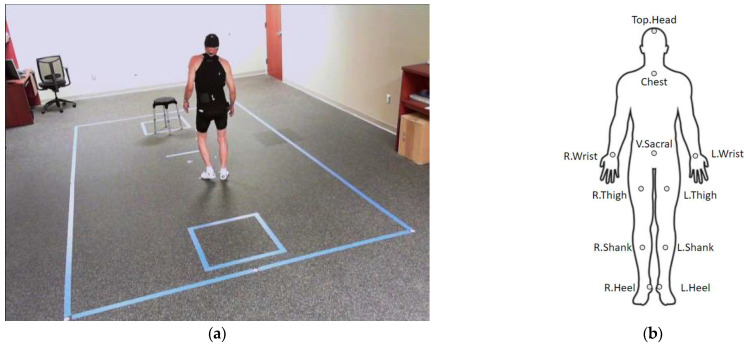
Motion capture recording in our lab. (**a**) Motion capture facility located in Emory Movement Disorders Clinic. (**b**) Marker sets used for analysis cover full-body kinematics.

All motor examinations were recorded using a 3-dimensional optical motion capture system (Motion Analysis Corporation, Santa Rosa, CA, USA). The motion capture facility is located at the Emory Movement Disorders Clinic. The capture area is 3.0 m *×* 4.6 m with 14 Osprey cameras having a resolution of 640 *×* 480 at 120 fps. Study participants were instrumented with tight-fitting clothes with 60 reflective adhesive markers configured as a superset of Helen Hayes kinematic markers set [37]. Figure 1 shows an example of a data capturing session with our motion capture system (Figure 1a) and the demonstration of the marker locations that are specifically used for analysis in this study (Figure 1b). Out of 60 markers, we used 11 markers corresponding to joint locations of the head, chest, sacral, left and right wrists, left and right thighs, left and right shanks, and left and right heels for full-body kinematics analysis. The collected kinematic data were preprocessed manually to correct erroneous marker trajectories by study staff, and missing data (2–5% of samples) were filled by linear interpolation and extrapolation. The preprocessed kinematic data were projected to a hip-centered coordination system and applied with Z-score normalization for our analysis. 

### 2.2. Experiments 

#### 2.2.1. PD Subtype Calculation 

As our goal is to identify PD motor subtypes from full-body kinematic time-series data, we calculated the corresponding subtypes of each participant from MDS-UPDRS parts II and III following standard practice [5]. First, a tremor-related components score was calculated from the average of MDS-UPDRS items 2.10, 3.15, 3.16, 3.17, and 3.18 (tremor, postural and kinetic tremor of the hands, rest tremor amplitude, constancy of rest tremor). A gait-related components score was calculated from the average of MDS-UPDRS items 2.12, 2.13, 3.10, 3.11, and 3.12 (walking and balance, freezing, gait, freezing-of-gait, postural stability). Then, the score ratio was computed by dividing the tremor-related components score by the gait-related components score. The patients with the ratio ≤0.90 and ≥1.15 were determined as PIGD and TD subtypes, respectively. Otherwise, patients’ subtypes were classified as indeterminate. Due to a lack of sufficient data from the Indeterminate subtype, we excluded patients’ data with the Indeterminate subtype from our analysis, eventually having N = 55 for our analysis. Table 2 shows the overall statistics of our participants with respect to PD motor subtypes and FOG scores (MDS-UPDRS 3.11 item). The overall dataset shows a highly skewed class distribution having 82% for the PIGD subtype, which is also known to be more prevalent in parkinsonism [5,7]. 

#### 2.2.2. Overall Classification Pipeline

All participant sessions were assigned labels for the corresponding PD motor subtype. Then, for the experiment, we applied a standard human activity recognition pipeline using a sliding window approach [39]. The preprocessed 3D kinematics time series from 11 markers were segmented with a 4-s window with 1-s overlaps. We adopted a 4-s window size, which was studied to be effective for movement analysis for PD in previous studies [40]. We extracted gait, statistical, or frequency feature representations from each analysis window, which we will describe shortly. Then, extracted features were used to train with various machine learning models widely used in previous studies in PD analysis, including Random Forest (RF) [41,42], Support Vector Machine with radial basis function kernel (rbfSVM) [43,44], and multilayer perceptron (MLP) [45,46,47]. 

#### 2.2.3. Gait Features 

As a baseline experiment, we studied gait parameters to classify PD subtypes, which were frequently used in previous studies [6,22]. Following previous work, we extracted gait parameters for both the left and right sides, including step length average (cm), stride length average (cm), forward velocity average (cm/s), cadence average (steps/min), total support time (%), swing phase (%), initial double support time (%), single support time (%), step width (cm), range and average of sagittal-plane knee and ankle angles during the gait cycle (°), where we only considered the sagittal plane as our kinematic marker set was not designed for frontal/rotation angles, from Walk-Thru testing sessions. Most Walk-Thru testing sessions were shorter than 4 s window, so we extracted summarized gait features over the entire sequence in each session, consisting of 120 gait samples from 55 study participants. The collected gait parameters were Z-standardized for the experiment. The standardized gait parameters were used for model training and prediction as described earlier. We also iterated analysis with step lengths normalized to participant heights following standard practice to omit the effect of height. Height information was available for 43/55 participants; for the remainder, the mean height (180 cm) was imputed. 

#### 2.2.4. Full Body Kinematics Features 

Here we describe our proposed method for classifying 11 3D kinematics time series from three different sessions (Walk-Thru, Turn-360°, and TUG) into PD subtypes. From each 4-s analysis window, we extracted statistical and temporal-frequency features that are known to be useful to characterize PD movements from previous works [48,49,50]. For statistical features, we extracted mean, minimum, maximum, and variance. For temporal-frequency features, we extracted freezing index (fi) [51], central frequency (cenfreq), dominant frequency (domfreq), and mean of wavelet coefficients (wav) [52] features from each X, Y, Z channel of 11 markers. The collection of features was concatenated and Z-standardized for model training and testing. In the following analysis, features will be referred to by their abbreviated form. For example, “fi-L.Thigh_Z” refers to the freezing index feature computed from the Z channel of the left thigh marker. 

#### 2.2.5. Model Evaluation 

We validated both gait and full-body kinematics features explained above for classifying PIGD and TD for all three session types (TUG, Walk-Thru, and Turn-360°). Furthermore, we also studied the effect of removing FOG for identifying PD motor subtypes by including or excluding patients with FOG in our experiments [38]. Since FOG is more prevalent in patients with PIGD subtypes, this is to investigate how our model can generalize for the movements in PIGD subtypes when present without FOG. 

For all cases, we evaluated both user-dependent and user-independent cross-validations to simulate a scenario where the same patient data in the testing set are available for the training set or not. For the user-dependent model, data from the same patients were used for both training, validation, and testing set. We avoided including analysis windows adjacent to the training set into the validation or test set to remove pairwise similarity biasing the cross-validation results [53]. For the user-independent model, different sets of participants were selected for training, validation, and testing. For both user-dependent and independent cross-validation, we used 50%, 20%, and 30% of the dataset for the training, validation, and testing sets, respectively, at each fold. Specifically, we applied repeated 5-fold cross-validation (50 repeats) to evaluate PD subtypes classification performance to consider randomness in model training processes. The model hyperparameters were optimized using a single run of nested 5-fold cross-validation with randomized parameter search, which was used across all 50 repeated runs in our evaluation [54]. 

For performance metric, we used binary F1 score, which is a harmonic mean of precision and recall, that is widely used in measuring classification performance [27]:

(1) $$\mathrm{Precision}=\frac{TP}{TP+FP}$$

(2) $$\mathrm{Recall}=\frac{TP}{TP+FN}$$

(3) $$F1\;\mathrm{score}=2\;\times \;\frac{Precision\times Recal}{Precision+Recal}$$

*TP* is a true positive that represents the total of successfully classified class windows, *FP* is a false positive that represents the total misclassified class windows, and *FN* is a false negative that represents the total misclassified non-class windows. For evaluating statistical significance, we used a Wilson score interval for a repeated (50 repeats) 5-fold cross-validation (N = 250) [55]. 

#### 2.2.6. Potential Biases in Classification Performance 

For both user-independent and user-dependent cross-validation, we assessed the potential biases associated with different models (RF, rbfSVM, and MLP), different features (gait or kinematics), different sessions (Walk-Thru, Turn-360°, and TUG), and patient characteristics (with and without FOG). After computing individual F1 scores for all possible combinations of models, features, sessions, and patient characteristics, we statistically compared model performance using linear mixed-effects model analysis [56]. Based on the best combination, we further analyzed potential bias in model performance associated with patient demographics, which are FOG-included, PD duration, age, and sex. Specifically, after computing individual F1 scores for each participant, the linear mixed-effects model used dichotomized age (ages < 69 and ≥69), FOG, and sex as predictors of individual F1 score. Statistical significance was assessed with Wald tests at *p* = 0.05. 

#### 2.2.7. Explaining Kinematic Features with Feature Analysis 

Based on the classification experiments, we studied how different features were affecting classification scores. In our experiment shown in the following section, TUG sessions with kinematic features and RF model produced the highest classification performance in user-dependent experiments, where we analyzed the importance of each feature from kinematic data for classifying PD motor subtypes. Specifically, following the standard practices for a random forest model, we sorted feature importance based on the accumulation of the impurity decreases within each tree [57]. Mean decrease in impurity (MDI) scores were obtained for each feature by computing the mean decrease in impurity ($1-\sum_{i}^{c}p_{i}^{2}$, where c is the number of classes and *p_i_* is the probability of the feature being class *i*) introduced by the feature across an ensemble of trees during the construction of a random forest. Features that introduce a greater decrease in mean impurity are considered more important for separating classes. This is conducted for experiments for cases both including and excluding FOG samples for our classification task. 

For the most informative features determined from the feature importance analysis, we further conducted correlation analysis with respect to tremor- and gait-related scores that were computed from MDS-UPDRS-III. Specifically, we used the Pearson correlation coefficient to rank features computed from the TUG session with FOG samples. We note that the feature samples used for correlation analysis were not independent, as these features were calculated from 4-s analysis windows using overlapping sliding windows. 

## 3. Results 

To understand the utility of kinematic features for PD motor subtypes classification in a realistic deployment scenario, where a trained model is deployed for unseen users, we first studied a user-independent model experiment. Based on this analysis, we studied the effect of personalizing the model through user-dependent scenarios afterward. Overall results of our experiments are shown in Table 3 for user-independent cross-validation and in Table 4 for user-dependent cross-validation. 

### 3.1. User-Independent Model Using Gait and Kinematic Features 

#### 3.1.1. Gait Features 

We studied the effectiveness of gait features for user-independent scenarios in Table 3 (first and second rows). For classifying between PIGD and TD, gait features from Walk-Thru sessions with the RF model showed 61.0% F1 score (Table 3, first row) when including patients experiencing FOG and showed 59.5% F1 score (Table 3, second row) when without patients with FOG. For rbfSVM, the classification was over random chance (50% for binary classification task), showing 63.9% (including FOG) and 57.8% (excluding FOG). The MLP model achieved similar scores above random chance showing 53.0% (including FOG) and 64.2% (excluding FOG). When we iterated the analysis with step length normalized to height, the overall results were relatively insensitive to units of step length. The largest change in the F1 score was from 63.9% ± 6.0 to 64.9% ± 5.9 for the gait feature experiment that included FOG samples. 

#### 3.1.2. Full Body Kinematics Features 

For the Walk-Thru session when using RF (Table 3, third and fourth rows), kinematic features improved by an absolute 5.5% F1 score when including FOG samples and an absolute 2.9% F1 score when excluding FOG samples. When using data from patients without FOG, kinematics features showed a 79.6% F1 score when using rbfSVM (Table 3, fourth row), significantly outperforming the gait feature by an absolute 21.8% F1 score. PD motor subtype classification was also very effective when using Turn-360°sessions (Table 3, fifth and sixth rows), showing a 77.6% F1 score with rbfSVM including patients with FOG. For the patients without FOG, RF models showed a 71.3% F1 score. When classifying TUG sessions with FOG samples (Table 3, seventh row), the best performance was shown for the rbfSVM model (69.1% F1 score), which was higher than classifying Walk-Thru sessions with the RF model (an absolute increase of 2.6%), but lower than classifying Turn-360° sessions with the rbfSVM model (an absolute reduction of 8.5%). Overall, for the user-independent scenario, Turn-360° sessions showed the highest performance (77.6% F1 score) for patients with FOG, and for patients without FOG, Walk-Thru sessions showed the highest performance (79.6% F1 score). 

#### 3.1.3. Potential Biases in Classification Performance 

Our classification results in Table 3 differ based on different models (RF, rbfSVM, and MLP), different features (gait or kinematics), different sessions (Walk-Thru, Turn-360°, and TUG), and patient characteristics (with and without FOG). The linear model found no significant differences in F1 scores including patients with FOG, as also demonstrated in the Wilson score interval analysis in Table 3. As expected, kinematic features showed an overall improvement of an absolute 20.1% F1 score increase, compared to gait features (*p* < 0.001). Across different models, the RF and rbfSVM models showed absolute 9.6% (*p* < 0.001) and 9.2% (*p* < 0.001) improvements compared to the MLP model, respectively. Additionally, the Turn-360° sessions resulted in an overall improvement of an absolute 3.9% (*p* < 0.001) compared to the TUG sessions. Our model found no significant difference in F1 scores between TUG and Walk sessions. 

Using the best combination of model and features from the user-independent experiments (rbfSVM, Turn-360°), we analyzed the potential bias associated with patient demographics. We found no significant differences in F1 scores for demographic factors of age, sex, PD duration, or FOG inclusion. 

### 3.2. User-Dependent Model Using Kinematic Features 

Observing significant improvement in the classification rate using kinematic features from user-independent models, we studied further into kinematic features when the model is personalized to each patient. Walk-Thru sessions including FOG samples improved the absolute 25.4% F1 score (Table 4, first row) for the RF model when compared to the best performance for the user-independent setting. For the sessions without FOG samples, the RF model showed a 94.1% F1 score (Table 4, second row), which improvement was an absolute 14.5% F1 score compared with the best performance for a user-independent setting using rbfSVM. For the Turn-360° session including FOG samples, the best classification performance, was 86.0% with rbfSVM (Table 4, third row), which had an absolute increase of 8.4% F1 score from the corresponding results for a user-independent setting. For the experiment without FOG data, the RF model showed 92.3% (Table 4, fourth row) for Turn-360 sessions, having an absolute improvement of 21.3% F1 score from the user-independent session. Surprisingly, for TUG sessions (Table 4, fifth and sixth rows) the user-dependent model was extremely effective, showing 88.7% and 95.4% for the experiment using the RF model with and without FOG samples, which corresponds to an absolute increase of 19.6% and 23.3% from the best user-independent results, respectively. 

#### Potential Biases in Classification Performance 

Likewise in the user-independent experiment, we studied potential biases in model performance associated with models, features, sessions, and FOG. The linear model found excluding FOG samples makes a statistically significant difference, increasing the F1 score by absolute 2.8% (*p* < 0.001). This differed from the user-independent case, where FOG samples did not have significant differences. Compared to MLP, both RF and rbfSVM had statistically significant improvements, showing absolute increases of 8.7% and 5.5% F1 scores overall (both *p* < 0.001), respectively. Additionally, the TUG sessions resulted in an overall improvement of an absolute 2.9% (*p* < 0.001) compared to the Turn-360°sessions. Our model found no significant difference in F1 scores between TUG and Walk sessions. 

We also analyzed the best combination of model and features (RF, TUG) for potential bias from demographic factors for the user-dependent model. As with the user-independent model, we found no significant difference in F1 scores for demographic factors of age, sex, PD duration, or FOG inclusion. 

### 3.3. Feature Relevance from Kinematics Features 

#### 3.3.1. Most Informative Features for Classification 

**Figure 2:**
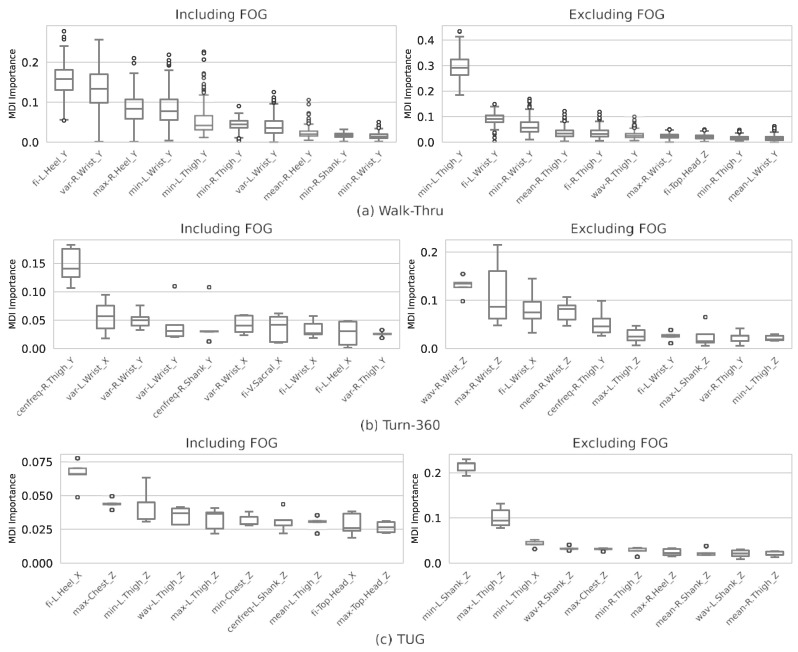
Most important features by mean decrease in impurity (MDI) importance scores from user-dependent RF model trained on each testing session. Figures on the left include important features from experiments including FOG samples and figures on the right include important features from experiments only considering samples with a FOG score of 0. Important features from all three experiment types are shown: (**a**) Walk-Thru, (**b**) Turn-360°, and (**c**) TUG.

Figure 2 presents the top ten most informative features from TUG, Walk, and Turn-360° sessions with a user-dependent RF model. For Walk-Thru experiments, when FOG is included (Figure 2a, left), top features include fi-L.Heel_Y, variance-R.Wrist_Y, maximum-R.Heel_Y, minimum-L.Wrist_Y, and minimum-L.Thigh_Y. When FOG is not included (Figure 2a, right), top features include minimum-L.Thigh_Y, fi-L.Wrist_Y, minimum-R.Wrist_Y, mean-R.Thigh_Y, and fi-R.Thigh_Y. 

For Turn-360°experiments, when FOG is included (Figure 2b, left), top features include cenfreq-R.Thigh_Y, variance-L.Wrist_X, variance-R.Wrist_Y, variance-L.Wrist_Y, and cenfreq-R.Shank_Y. When FOG is not included (Figure 2b, right), top features include wavR.Wrist_Z, maximum-R.Wrist_Z, fi-L.Wrist_X, mean-R.Wrist_Z, and cenfreq-R.Thigh_Y. For TUG experiments, when FOG is included (Figure 2c, left), top features include fi-L.Heel_X, max-Chest_Z, minimum-L.Thigh_Z, wav-L.Thigh_Z, and maximum-L.Thigh_Z. When FOG is not included (Figure 2c, right), top features include minimum-L.Shank_Z, maximum-L.Thigh_Z, minimum-L.Thigh_X, wav-R.Shank_Z, and max-Chest_Z.

#### 3.3.2. Feature Correlation Analysis 

We further examined the top 10 most relevant features from TUG, which showed the highest classification scores from Table 4, with respect to the tremor- or gait-related components score. Examining the top 10 most relevant features from the FOG-included experiment for correlation with the tremor-related components score, we found that fi-L.Thigh_X (freezing index) exhibited the strongest correlation at *r* = 0.117. This was followed by fi-L.Shank_X (*r* = 0.095) and fi-L.Heel_X (*r* = 0.047). Examining the top 10 most relevant features for the correlation with the gait-related components score, we found that cenfreq-L.Shank_Z had the strongest correlation at *r* = 0.135. This was followed by min-L.Thigh_Z (*r* = 0.113), and min-R.Thigh_Z (*r* = 0.111). 

For the feature correlations when excluding FOG samples, wav-R.Shank_Z exhibited the strongest correlation with the tremor-related components score at *r* = −0.40, followed by mean-R.Shank_Z (*r* = −0.40) and min-L.Shank_Z (*r* = −0.39). When considering the correlations of these features with the gait-related components score, we see large correlations from features min-L.Thigh_Z (*r* = 0.45), wav-L.Thigh_Z (*r* = 0.44), and mean-L.Thigh_Z (*r* = 0.44). This stronger feature correlation explains higher classification scores when excluding FOG samples, as observed in our experiment results. 

#### 3.3.3. Analysis with Restricted Feature Set 

Using only the top-5 and top-10 features from the TUG user-dependent FOG-included experiment, we trained an additional random forest model to examine classification performance. The top-10 feature random forest achieves an F1 score of 0.867 *±* 0.041, a decrease of 0.014 from the model using a complete feature set. The top-5 feature random forest achieves an F1 score of 0.768 *±* 0.052, a decrease of 0.119 from the model using a complete feature set. 

#### 3.3.4. Analysis with Restricted Marker Set 

We also tested classification performance when using only features computed from the markers represented in the top 10 features. From the TUG user-dependent FOG-included experiment (Figure 2c, left), we obtained Thigh and Shank as the top markers. We trained a random forest model on a complete feature set computed from the left and right thigh and shank markers which achieved an F1 score of 0.895 *±* 0.037, an increase of 0.008 from the model using all markers. 

## 4. Discussion 

### 4.1. User-Independent Model Using Gait and Kinematic Features 

#### 4.1.1. Gait Features 

The result was surprising, as previous work using statistical analysis showed potential for using gait features to identify PIGD and TD [6,22]. Our results show that despite statistical significance, gait features are not discriminative when it comes to distinguishing PIGD and TD. Also, considering the confidence interval, patients experiencing FOG did not significantly affect classification performance. Overall, gait features across entire testing sessions do not appear to be sufficiently reliable to predict motor subtypes. 

#### 4.1.2. Full Body Kinematics Features 

Compared to gait features, full-body kinematic features significantly improved performance in classifying PIGD and TD, showing the importance of incorporating whole-body movement when analyzing an entire gait cycle. Interestingly, classifying TUG sessions showed mixed performance compared to Walk-Thru and Turn-360° sessions (Table 3, seventh and eighth rows), although the TUG session included walking and turning, capturing both movements in Walk-Thru and Turn-360°. It showed the opposite behavior when classifying TUG sessions excluding FOG samples (Table 3, eighth row). The best performance was shown when classifying with rbfSVM (72.1% F1 score), which was lower than classifying the Walk-Thru session with rbfSVM (an absolute reduction of 7.5% F1 score), but also higher than classifying Turn-360° sessions with RF models (an absolute increase of 0.8%). We consider this mixed outcome came from two reasons: (1) the significance of individual differences due to the complexity of TUG sessions and (2) differences in movement characteristics for the patients with or without FOG. For unseen patients, while walking and turning, the trained model has difficulty generalizing to account for variations in individual characteristics. 

#### 4.1.3. Potential Biases in Classification Performance 

RF and SVM significantly outperformed the MLP. We consider that the improvement of the RF model came from highly flexible non-linear decision boundaries learnable from ensemble decision trees and that the improvement of the rbfSVM model came from the kernel method that can project kinematic features to linearly separable, high-dimensional feature spaces. Although MLP models can model non-linear boundaries, the model performance can be significantly affected by non-optimal local minimums. 

### 4.2. User-Dependent Model Using Kinematic Features 

In Table 4, we demonstrate the effect of a user-dependent model, which learns the movement patterns of testing patients from training datasets for the same patient. Learning the individual characteristics of patients could significantly improve the model performance compared to the user-independent models. These results show the importance of developing personalized models, as the movement symptoms of individuals vary significantly in parkinsonism [58]. Especially, when excluding FOG samples, we consider a high classification rate (95.4%) to account for the well-balanced class samples in our dataset. Shown in Table 2, 82% of data is for the PIGD subtype when including all patient samples experiencing FOG (FOG score > 0). But, when only considering samples with FOG = 0, we have nearly equal sample sizes between PIGD and TD samples. Overall, the TUG session combining walking and turning showed the highest classification rate when the model could learn the complex movements from the patients directly. 

#### Potential Biases in Classification Performance 

The RF model performed significantly better than rbfSVM in the user-dependent case. We consider this came from RF being capable of learning highly complex decision boundaries compared to rbfSVM, as rbfSVM essentially requires the feature space to be linearly separable after applying the kernel method, which limits its flexibility. For user-dependent cases, it is important for a model to learn decision boundaries that are personalized for individuals, which can be highly variable depending on the patient. On the other hand, user-independent models focus on learning decision boundaries that can generalize to unseen patients. 

### 4.3. Feature Relevance from Kinematics Features 

#### 4.3.1. Most Informative Features for Classification 

Across different sessions, lower-body movements, such as thigh or shank, had the highest importance. Interestingly, when only considering a single mode of movement, either walking (Walk-Thru) or turning (Turn-360°), upper body markers, like wrists, were among the top ten important features as well, indicating the potential of using wrist wearable sensors for assessing PD subtypes. For TUG experiments considering movements in both walking and turning and their transitions, the most informative features generally come from the left side of the lower body. This is likely due to the path taken in the TUG, which requires participants to make only left turns in our experiment, potentially emphasizing the difference in motor impairment between subtypes concerning the left side of the body. 

#### 4.3.2. Feature Correlation Analysis 

This analysis shows that the features useful for classifying between PIGD and TD have a moderate correlation with the gait-related components score and the tremor-related components score. Yet, the tremor-related components score had more correlation with freezing indices (fi) from lower body parts, which is known to be useful for capturing tremulous episodes in PD [51]. On the other hand, PIGD had more correlation with the size of movements involved in walking that is measured by statistical features, which is also well represented in posture instability and gait difficulty in PD. Thus, these associations may warrant further investigation for further interpretations. 

#### 4.3.3. Analysis with Restricted Marker Set 

These results show the potential to deploy a few sets of wearables that only capture shank and thigh movements to assess PD subtypes in ambulatory settings, significantly lowering the patient burden to go through full-body marker-based motion capture analysis. 

### 4.4. Utility of Gait Features 

Our results demonstrate that lower-body kinematic features are more capable of reliably distinguishing PD motor subtypes than gait features alone. We observed an absolute 22.3% increase in F1 score relative to the gait-feature baseline of 57.3%. This result is consistent with the finding from Herman et al. [6] that some gait parameters, such as gait speed, stride length, and variability, do not differ between PIGD and TD groups. However, others also found statistical differences in stride length, stride time, and variability between motor subtypes, demonstrating conflicting opinions on gait parameters for distinguishing PIGD and TD [22]. Our analysis using ML, additionally considering FOG, draws a conclusion that gait parameters are insufficient to discriminate between PIGD and TD, and that full-body kinematic features are more useful. 

### 4.5. Utility of Kinematics Features 

Our results are consistent with the findings in Rezvanian et al. [23] that frequency components can be used to differentiate motor subtypes, as we found wavelet coefficient mean, central frequency, and freezing index among the most important features (Figure 2). However, it may not be directly comparable due to differences in the recording paradigm, as the previous study collected the standing center of pressure (COP) measure from a static test as opposed to kinematics data from dynamic walking tasks. 

### 4.6. Limitations 

One limitation of this study is the imbalance of motor subtypes among participants, as PIGD consists of 82% in the FOG-included condition. This label imbalance presented a challenge to classification models when recognizing the TD subtype. Disease duration also contributes to the imbalance, as PIGD is associated with functional disability that commonly increases during the progression of PD [7]. Many of this study’s participants have a disease duration of several years, which explains the prevalence of PIGD, as a majority of them are in the stage of advanced PD, experiencing FOG including H&Y stage III and IV, and having an MDS-UPDRS-III Score of 34.7 ± 10.5 and disease duration of 7.5 ± 4.5 years, as shown in Table 1. In our future study, we plan to recruit more patients to conduct a larger scale dataset with a balanced amount of each motor subtype, also including intermediate subtypes and early and late stages of PD to study the generalizability of our findings in this paper in a more diverse population. 

Finally, another potential limitation is that due to the nature of the dataset used, we cannot evaluate the extent to which inter-subject error in marker placement or marked anthropometric/morphological differences between patients might influence results. We found no significant difference in F1 scores for demographic factors, suggesting that the influence of this potential problem is minimal; however, more data are needed to address this question. 

Our decision on using statistical and temporal-frequency features with a 4-s window size was based on previous work in PD analysis [27]. Some work concerning the prediction of freezing of gait has tested multiple sliding window durations from 2 s to 4 s with a 0.5-s overlap and also suggested using a window duration of 2–2.5 s, but those differences in performance were not statistically significant [49]. Had we optimized sliding window size and feature sets, we would have potentially improved classification performance, but our goal was not to provide a specific recommendation on hyperparameters. Instead, we controlled those parameters to be similar across experiments to demonstrate the importance of having full-body kinematics data for identifying PD motor subtypes, which was also successful with our experiment setting. 

### 4.7. Future Directions 

This study was conducted with a marker-based motion capture system to collect a 3D kinematics dataset. Although our kinematics dataset can measure high-fidelity (millimeter-level) movements, it cannot be deployed in the real world to track continuous movements associated with PD motor subtypes. The movement symptoms in PD change according to medication cycles, and it is critical to understand individual differences in how patients with varying motor subtypes are affected with respect to their quality of life [8]. Our findings suggest that movement assessment in ambulatory settings is possible as minimal behavioral testing instrumentation, for example, using wearables for only thigh and shank locations, can sufficiently capture useful features from the lower body movements. In our future research, we will also investigate how findings from this work are generalized in real-world experiences of PD through the lens of computer vision techniques such as pose estimation [59]. Recent work has reported success with pose analysis using computer vision techniques to analyze movements in PD [60], avoiding the need for expensive motion capture equipment and special facilities and enabling the low-cost deployment of the system in patients’ homes. Furthermore, with large-scale video datasets collected from the real world, deep learning methods could also be used to automatically generate more informative representations of motion data, potentially improving upon feature engineering approaches [61]. 

Use of motor subtyping for PD is not without controversy. Previous studies have shown the TD and PIGD subtypes to have substantial variability, also suggesting that the akinetic-rigid (AR) motor subtype may also need to be considered. PIGD and TD subtypes showed significant overlaps in motor phenotypes, with many patients switching from one subtype to the other in both the Parkinson’s Progressive Markers Initiative (PPMI) and the University of Maryland Parkinson Disease and Movement Disorders Center (UM-PDMC) longitudinal dataset [62,63]. Ambiguous terminology, lack of reproducibility of the PIGD subtype in cluster analyses, the influence of age and comorbidities, and lack of unique clinical courses have all been cited as limitations of PD motor subtyping [64]. Yet, the validity of motor subtype classification is important for understanding the progression of PD, necessitating new, more robust approaches toward subtyping. 

Future work may investigate the use of unsupervised machine learning methods for data-driven PD motor subtype discovery. Clustering methods like unsupervised hierarchical clustering (HCA), KMeans, and random forest clustering have been used in previous studies to identify subtypes of PD [65,66,67]. One previous study identified five distinct motor subtypes of PD by using motor assessment scores from the Parkinson’s Progressive Markers Intitiative (PPMI) [10], but no previous studies have attempted to use full-body kinematics data with unsupervised methods to identify PD motor subtypes. To identify consistent and distinct motor subtypes over the course of PD, this approach would likely require the collection of longitudinal kinematics data. Unsupervised methods may help identify more consistent and valid PD motor subtypes, the classification of which is a key issue in clinical research on understanding the progression of PD. 

## 5. Conclusions 

Identifying PD motor subtypes helps to understand the prognosis of patients and to implement appropriate treatment plans that are personalized to individuals. Conventional practice for identifying PD motor subtypes is through standard movement assessment procedures (MDS-UPDRS), in which patients need to be manually examined by a movement disorder specialist. An automated system for subtyping may be more impactful in a research setting, where consistent subtyping is useful. To assist with efficiently assessing PD conditions, this study demonstrates a system that can identify PD motor subtypes objectively and automatically by using machine learning models and full-body kinematics data while patients walk. Our experiment demonstrated that a machine learning model with kinematic features could identify PD motor subtypes with 79.6% F1 score (user-independent) and 95.4% F1 score (user-dependent) for 55 patients with parkinsonism. Our feature analysis further showed that both temporal spectral and statistical features from lower body markers are informative in distinguishing motor subtypes. Additionally, our analysis of a restricted feature set suggests that we can use as few as two unique markers (thigh and shank) and still reliably distinguish motor subtypes. With these successful results, we plan to improve our model with a large-scale dataset and also study the generalizability of our findings using video-based technology that uses pose estimation techniques in the wild. With the current work and future plans combined, we expected to significantly decrease the cost of movement assessments to improve patient care for PD treatments. 

## Figures and Tables

**Table 1:** Clinical and demographic features of study participants. (LED: Levodopa Equivalent Dose, NFOG-Q: New Freezing of Gait Questionnaire).

	PD-FOG	PD-NoFOG	PP-FOG
**N**	35	17	5
**Age, y**	69 ± 7	67 ± 12	66 ± 6
**Sex**			
Male	30	11	2
Female	5	6	3
**Disease duration, years**	10.5 ± 6.7	6.0 ± 3.6	6.0 ± 3.3
**LED, mg**	1429 ± 673	833 ± 303	1258 ± 640
**MDS-UPDRS-III (OFF)**	34.0 ± 10.6	30.8 ± 13.2	39.4 ± 7.8
**Hoehn and Yahr Stage (OFF)**			
II	24	16	3
III	6	1	1
IV	6		2
**NFOG-Q**	20.1 ± 4.9	0.0 ± 0.0	17.8 ± 7.5

**Table 2:** Distribution of PD subtypes for 56 patients in our study in the “OFF” medication state. The PD subtypes are derived using MDS-UPDRS test based on standard practice as previously shown [5]. We also show the FOG score of the corresponding patients, which is known to be more associated with PIGD phenotypes [38]. In our dataset, one patient with TD subtype showed FOG with a FOG score of 1. We removed the Intermediate (Ind.) subtype for our analysis due to lack of sufficient dataset.

PD Subtype	FOG Scores					Total
	0	1	2	3	4	
PIGD	9	6	13	9	7	44
TD	10	1				11
Ind.	1					1

**Table 3:** Classification performances of a user-independent model. **Bold** means the best performance among RF, rbfSVM, and MLP.

Features	Testing Session	FOG Included	Mean F1 Score [%]		
			RF	rbfSVM	MLP
Gait	Walk-Thru	Yes	61.0 *±* 6.0	**63.9 *±* 6.0**	53.0 *±* 6.2
		No	59.5 *±* 6.1	57.8 *±* 6.1	**64.2 *±* 5.9**
Kinematic	Walk-Thru	Yes	**66.5 *±* 6.4**	66.0 *±* 6.4	62.8 *±* 6.5
		No	62.4 *±* 6.6	**79.6 *±* 5.5**	72.9 *±* 6.1
Kinematic	Turn-360°	Yes	67.3 *±* 6.1	**77.6 *±* 5.4**	69.9 *±* 6.0
		No	**71.3 *±* 6.2**	70.5 *±* 6.2	64.4 *±* 6.5
Kinematic	TUG	Yes	64.1 *±* 6.4	**69.1 *±* 6.1**	63.5 *±* 6.4
		No	66.3 *±* 6.5	**72.1 *±* 6.2**	68.4 *±* 6.4

**Table 4:** Classification performance for a user-dependent model. **Bold** means the best performance among RF, rbfSVM, and MLP.

Features	Testing Session	FOG Included	Mean F1 Score [%]		
			RF	rbfSVM	MLP
Kinematic	Walk-Thru	Yes	91.9 *±* 3.3	**92.6 *±* 3.1**	75.7 *±* 5.3
		No	**94.1 *±* 2.8**	89.9 *±* 3.7	81.3 *±* 4.8
Kinematic	Turn-360°	Yes	70.9 *±* 5.6	**86.0 *±* 4.2**	51.7 *±* 6.1
		No	**92.3 *±* 3.2**	89.3 *±* 3.8	77.5 *±* 5.1
Kinematic	TUG	Yes	**88.7 *±* 3.8**	74.7 *±* 5.3	84.5 *±* 4.4
		No	**95.4 *±* 2.5**	88.8 *±* 3.8	92.0 *±* 3.3

## Data Availability

The deidentified raw data and code for supporting the evidence in this work will be made available by the corresponding author upon reasonable request.
